# Fabrication of porous-Ti6Al4V alloy by using hot pressing technique and Mg space holder for hard-tissue biomedical applications

**DOI:** 10.1007/s10856-021-06546-2

**Published:** 2021-06-30

**Authors:** N. Aslan, B. Aksakal, F. Findik

**Affiliations:** 1 Department of Metallurgical and Materials Engineering, Faculty of Engineering, Munzur University, Tunceli, Turkey; 2 Munzur University Rare Earth Elements Application and Research Center, Tunceli, Turkey; 3 Department of Metallurgical and Materials Engineering, Faculty of Chemical and Metallurgy, Yildiz Technical University, Istanbul, Turkey; 4 Department of Metallurgical and Materials Engineering, Faculty of Technology, Sakarya University of Applied Sciences, Sakarya, Turkey

## Abstract

Porous-Ti6Al4V (P-Ti6Al4V) alloys were produced using the hot pressing and spacer methods for hard tissue biomedical applications and in particular, the effects of porosity on the mechanical and morphological properties of the structures were investigated. P-Ti6Al4V structures having the homogeneously distributed porosities at 41.08, 52.37 and 64.10% were fabricated by adding 40, 50 and 60% spherical magnesium (Mg) powder with 350 μm particle sizes in average as spacers and evaporating magnesium via the atmosphere-controlled sintering. The obtained porous structures were characterized by SEM, XRD and EDS. Furthermore, the strength and elastic modulus were evaluated by performing compression tests. Elastic modulus and densities were found to be 40–171 MPa, 2–5 GPa and 1.59–2.61, respectively and these values have been shown to decrease with an increase in porosity. The achieved density and mechanical property values, in particular, elastic modulus are close to human bone and within acceptable ranges for with biomedical application purposes. In addition, it was also found out from the analysis of produced P-Ti6Al4V that macropores were responsible for mechanical anisotropy contributed to formation of homogeneous and inter-connected open pores.

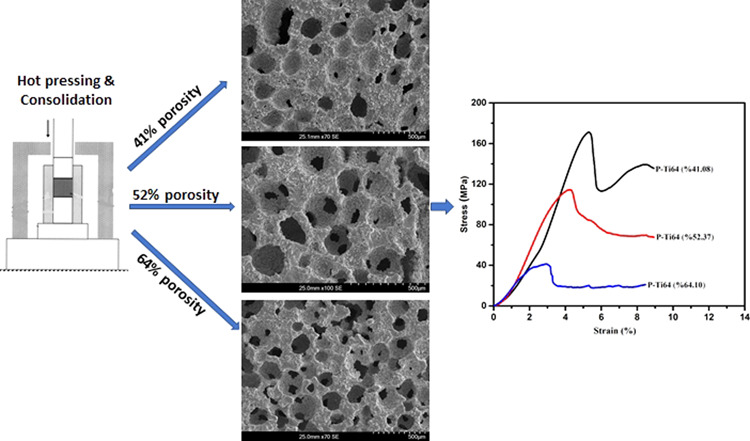

## Introduction

Among metallic materials, titanium and its alloys are considered to be the most suitable materials for biomedical applications due their properties such as biocompatibility, ease of shaping, perfect corrosion resistance, and low elastic modulus [1, 2]. However, the density and elastic modulus of titanium alloys are higher than that of human bone [3, 4]. This can lead to a stress shield and therefore, porous materials have been developed to reduce the elastic modulus of titanium-based alloys in order to approximate it to the value of natural bone [5–7]. These materials provide an advantage as the amount of material supporting the same cross-section area is less than bulk materials. In addition to low density and elastic modulus, porous materials can provide a better biological fixation/attachment by promoting bone tissue growth into pores that provide homogeneous stress transfer between implant and the bone [8, 9]. Furthermore, these structures can facilitate cellular activities such as transportation of nutrients and oxygen necessary for vascularization during bone tissue development [10, 11]. Mostly liquid and solid-state methods in powder metallurgy were used for production of metallic-based porous materials [12, 13]. Liquid state methods are widely used to produce Al, Zn, and Mg-based porous materials as they are easily processed thanks to their low reactivity and low melting points (660 °C). On the other hand, using such methods for the production of titanium and titanium-based porous alloys are very difficult to apply due to the high melting point and their excessive chemical affinity to atmospheric gases that rapidly dissolve above 400 °C. Methods called solid state or powder metallurgy can produce porous titanium alloys at much lower temperatures and under less chemical reactivity constraints, allowing more precise control of process variables and pore size [14].

The desired pore size, shape, volume, and distribution can be achieved by using a suitable space holding material especially in the production of titanium and titanium-based porous alloys [15]. Furthermore, it can protect titanium and its alloys from excessive contamination in terms of low temperature applicability with the space holder method. This process can be explained as mechanically pressing powders in room conditions with the help of a mold and sintering in an atmosphere-controlled furnace. In literature, ammonium hydrogen carbonate, carbamide, polymer, sodium chloride and magnesium particles have been used as a spacer in the production of porous titanium alloys [5–7, 16–22]. To the best of our knowledge, the powders were subjected to cold pressing and then sintering in those studies.

Unlike these studies, in this study, hot pressing technique and spherical Mg spacers where pressure and temperature are applied at the same time was used with improved consolidation between powders, P-Ti6Al4V alloys have been produced possessing more adequate mechanical properties with homogeneous and inter-connected open pores. The effects of different porosities on mechanical and morphological properties of the P-Ti6Al4V structures have been demonstrated.

## Experimental details

### Production of P-Ti6Al4V

**Fig. 1:**
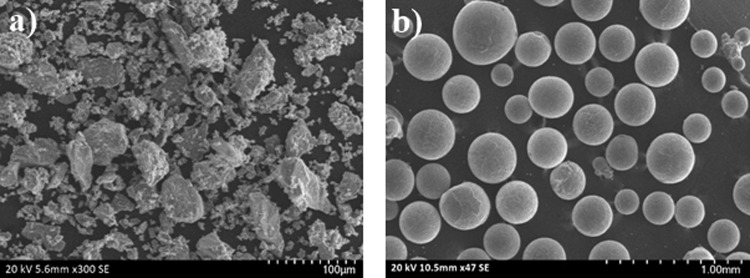
SEM views of **a** Ti6Al4V alloy powder and **b** Mg-space holder powder

Ti6Al4V alloy powder with 99.8% purity and 50-micron mean particle size (TLS Technik GmbH & Co. Germany) was used to produce P-Ti6Al4V alloys. Spherical magnesium powder with 99.8% purity and 150–850-μ particle size (350 μm in average) was used as spacers (Tang Shan Wei Hao Magnesium Powder Co., China). Figure 1a, b show the SEM images of the initial Ti6Al4V and Mg powders, respectively.

**Fig. 2:**
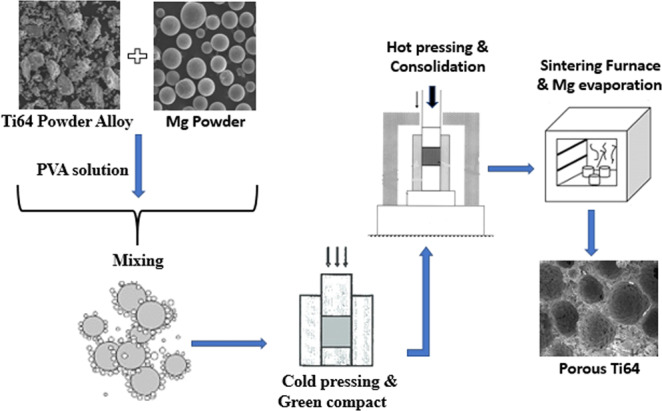
Production scheme of the P-Ti6Al4V alloy

In order to prepare the mixture, initially, the Ti6Al4V-Mg powder (40%, 50% and 60% in volume) was homogeneously mixed mechanically with 5% PVA (polyvinyl alcohol) fixing solution and then cold pressing was performed at 450 MPa with the help of a mold made of stainless steel (10 × 12 × 24 mm). After cold pressing, Ti6Al4V-Mg compact structures containing 40, 50, and 60% Mg were placed in 10 × 12 × 24 mm^3^ graphite molds, then they were subjected to a vacuum-controlled hot pressing unit (Celmak, Elazığ, Turkey) at 35 MPa pressure in vacuum atmosphere at 550 °C (below the melting point of Mg: 650 °C) for 10 min. Then, Mg that had been added to the structure was evaporated with the help of a tube furnace (MSE-furnace, 1500 °C Istanbul) under pure argon gas (99.999%), with 10 °C/min. heating-cooling speed and 2-h holding period at 1250 °C. During sintering, the evaporation temperature of Mg (1090°C) was taken into account and Mg created the aimed holes/pores in Ti6Al4V while moving away from the structure through evaporation at the end of the endothermic reaction. Such operations are illustrated schematically in Fig. 2.

### Density and Porosity Measurements

The density of the P-Ti6Al4V structures was measured based on Archimedes’ principle with a precision scale by selecting a xylol solution (CH_3_C_6_H_4_CH_3_) that has less density and faster inter-pore absorption than water [6]. In the first step, the P-Ti6Al4V samples were weighed in air and then they were submerged in a previously prepared xylol solution in a vacuum desiccator. They were kept under vacuum until the air bubbles were removed and the weight of the samples that absorbed xylol was measured. After weight measurements, the density (ρ) of the P-Ti6Al4V structures was calculated according to Eq. (1). The density of xylol (ρ_xylol_) was taken as 0.86 g/cm^3^.

1 $$\rho _{{\rm{porous}}} = \left[ {\frac{{m_{{\rm{porous,air}}}}}{{m_{{\rm{porous,air}}} - m_{{\rm{porous,xylol}}}}}} \right]x\rho _{{\rm{xylol}}}$$

where, *m*_porous,air_ represents the weight of P-Ti6Al4V in air, *m*_porous,xylol_ represents the mass of Ti6Al4V that absorbed xylol in xylol solution in vacuum environment, and *ρ*_xylol_ represents the density of the liquid solution.

The pore distribution properties and porosity percentages of P-Ti6Al4V structures were examined using a micrometric mercury porosimeter (AutoPore IV 9500 V1.09), which can apply pressure up to 0.52 psi at a contact angle of 130 degrees. A scanning electron microscope (Hitachi Su3500) and X-ray diffraction (Rigaku miniflex600) devices were used for morphological and structural characterizations. In addition, in order to characterize the stress-strain behavior of the P-Ti6Al4V structures, the compression strengths and elastic modulus were recorded by conducting compression tests at 1 mm/min. speed with a universal tensile tester (Shimadzu, 100 kN).

## Results

Results of morphology and mechanical properties of P-Ti6Al4V alloys produced in various porosity rates were presented in related Sections.

### P-Ti6Al4V Production, Density and Porosity

**Fig. 3:**
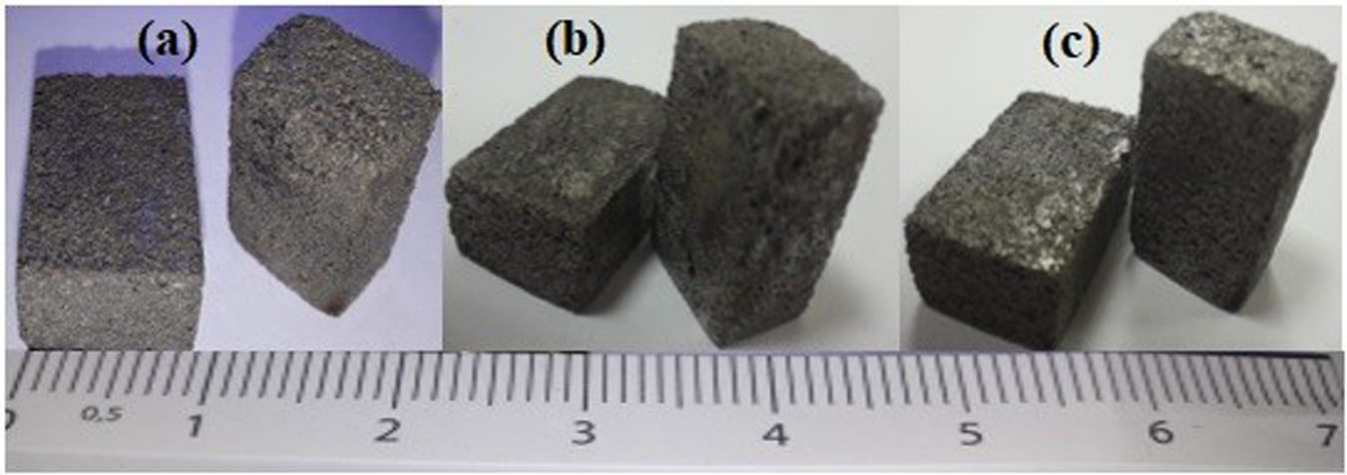
Images of **a** P-Ti6Al4V (41.08%), **b** P-Ti6Al4V (52.37%) and **c** P-Ti6Al4V (64.10%) structures

In hard tissue biomedical applications, it is always desired to have lower density and elastic modulus to obtain similarity with natural bone. For this aim, wide range of studies, such as producing porous and scaffold biomedical materials, are still being studied worldwide. Figure 3 shows the images of P-Ti6Al4V alloys with 41.08, 52.37, and 64.10% porosity and 2.61, 2.11, and 1.59 g/cm^3^ densities were obtained by subjecting Ti6Al4V-Mg compact structures with the addition of initial 40, 50 and 60% spherical magnesium spacers at 1250 ^o^C sintering under high-purity argon gas.

The density measurements of the P-Ti6Al4V structures were performed according to Eq. 1 and the results were summarized in Table 1.

**Table 1:** The density and porosity percentage of P-Ti6Al4V structures with different Mg proportions

Properties	Magnesium Additive (vol.%)		
	40	50	60
Density (ρ = g.cm^−3^)	2.61 ± 0.01	2.11 ± 0.015	1.59 ± 0.020
Porosity (%)	41.08 ± 0.22	52.37 ± 0.34	64.10 ± 0.47

### Morphological and structural characterization of P-Ti6Al4V

**Fig. 4:**
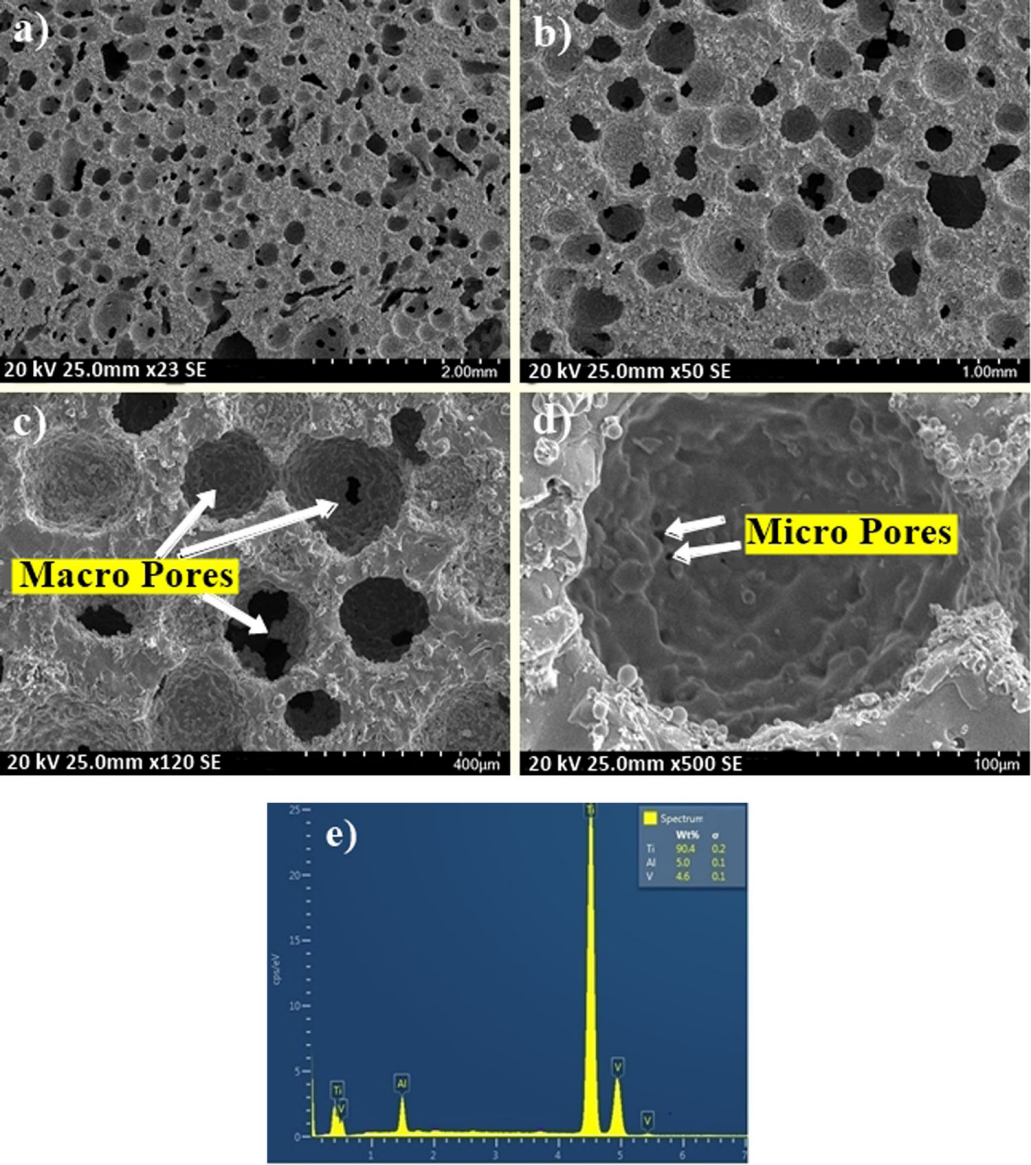
SEM images of P-Ti6Al4V (41.08%) samples at **a** ×23, **b** ×50, **c** ×120, **d** ×500 magnifications and **e** EDS spectrum

**Fig. 5:**
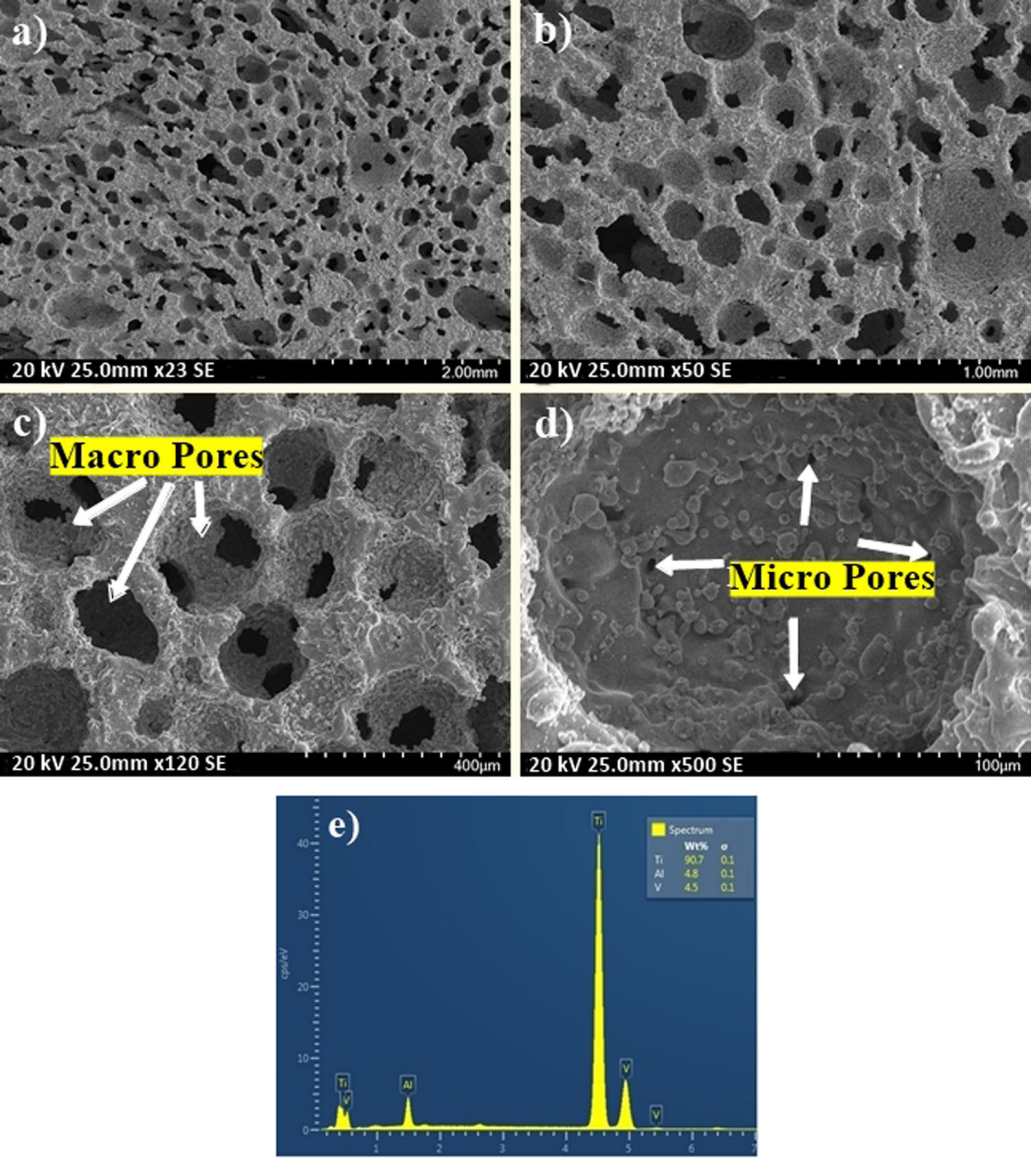
SEM images of P-Ti6Al4V (52.37%) samples at **a** ×23, **b** ×50, **c** ×120, **d** ×500 magnifications and **e** EDS spectrum

**Fig. 6:**
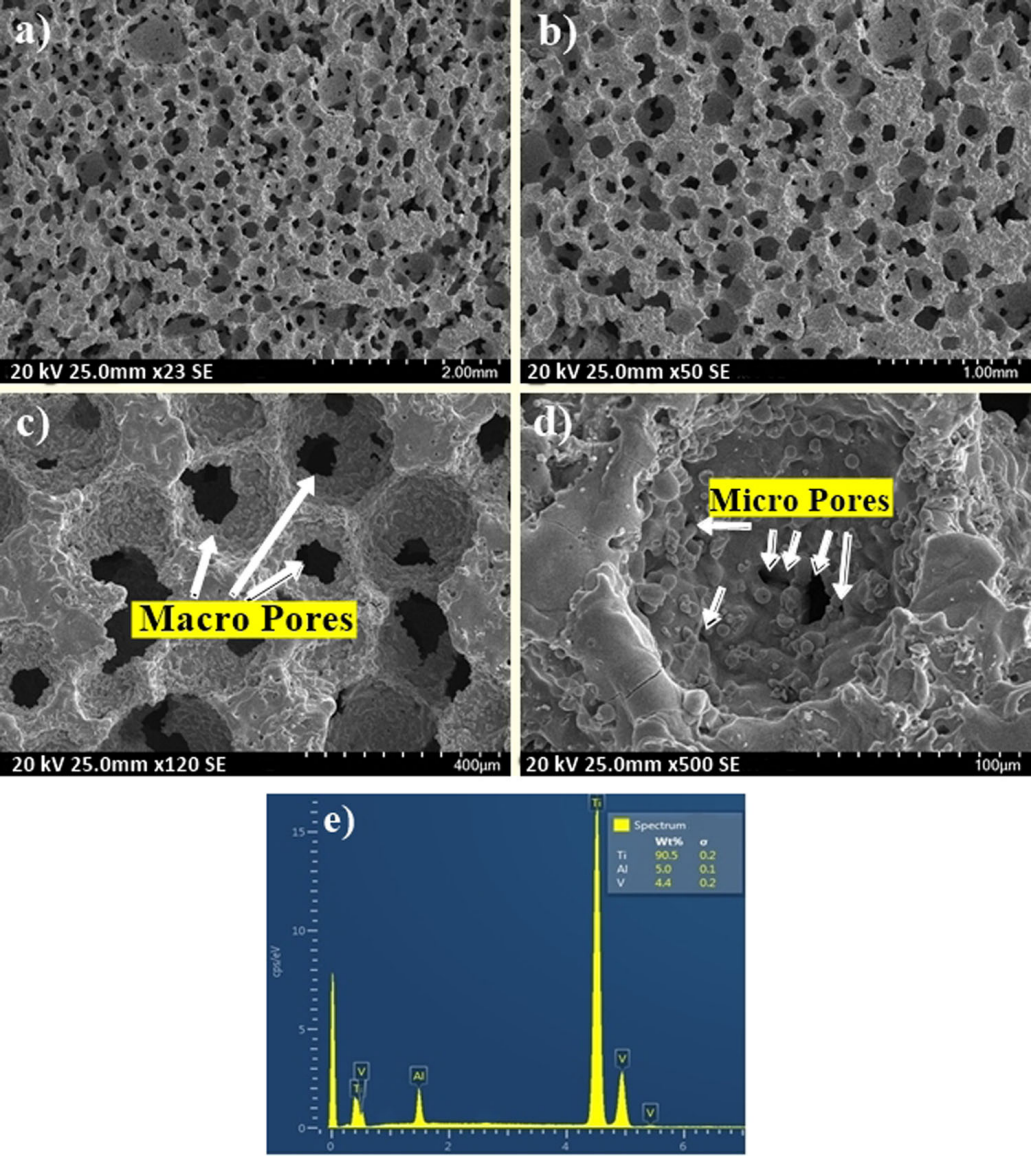
SEM images of P-Ti6Al4V (64.10%) samples at **a** ×23, **b** ×50, **c** ×120, **d** ×500 magnifications and **e** EDS spectrum

SEM images of the P-Ti6Al4V structures with 41.18, 52.37 and 64.10% porosity are shown in Figs. 4, 5, and 6, respectively. It is understood from these figures that the P-Ti6Al4V structures are distributed homogenously within almost spherical pores where the porous structures are connected to each other with a good network, and the macropores form closer walls as a result of hot pressing technique.

### Mechanical Tests

**Fig. 7:**
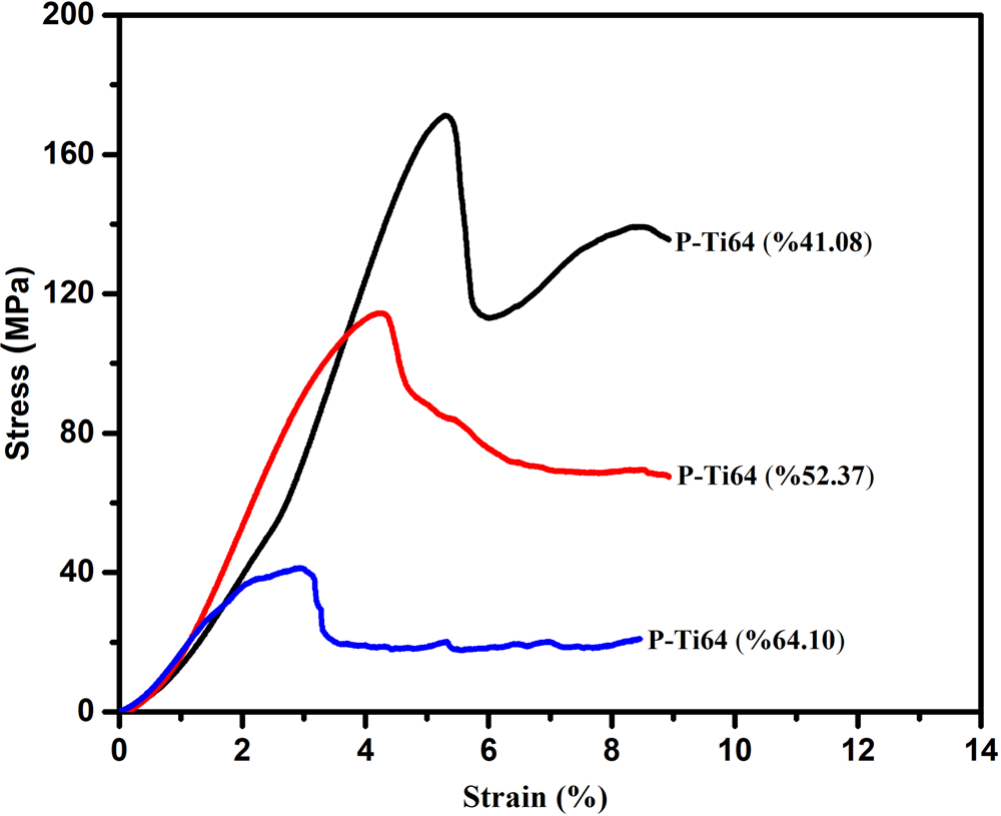
Stress–strain curves of P-Ti6Al4V structures with varying porosity percentages

Figure 7 shows the stress–strain curves of P-Ti6Al4V structures with porosity percentages of 41.08, 52.37 and 64.10%, and their compression strengths were determined as 171.86 ± 7.91, 113.54 ± 3.07, and 40.23 ± 1.52 MPa, respectively. The resulting curves show the typical characteristics of metallic foams that begin with linear elastic deformation and follow the plateau stage with a plain flow stress as the strain increases [23]. In this deformation zone, the pores compacted by the compression, and became more condensed as they cause a mass on the cell walls.

## Discussion

### Effect of Mg on Density

When the SEM images of produced P-Ti6Al4V structures given in Figs. 4–6 were examined, it is observed that the amount of porosity increased significantly with the increment of Mg content, and the structures contained two types of pores as macropores and micropores. Furthermore, SEM images indicates that macropores (100–400 µm) rather than micropores have formed by evaporation of Mg particles in which the obtained pores were directly affected by the size, shape and quantity of applied particles. On the other hand, micropores were also rarely observed on cell walls or cell edges between sintered powders. Because the reason for such micropores remained in the structure may be due to possible insufficient sintering or the fact that Mg particles which was evaporated during sintering broken off the small-sized Ti6Al4V powders by the use of the argon gas. Although the pore size requirement for bone growth towards porous structures was not fully defined in biomedical applications, and the macropore dimensions obtained in this study were found to be between the optimum size (100–500 µm) as reported in the literature [24–26]. Furthermore, from the EDS spectra of the P-Ti6Al4V structures, Ti:Al:V peak ratios were observed to range from 90.4 to 90.7, from 4.8 to 5, and from 4.4 to 4.6 respectively.

**Fig. 8:**
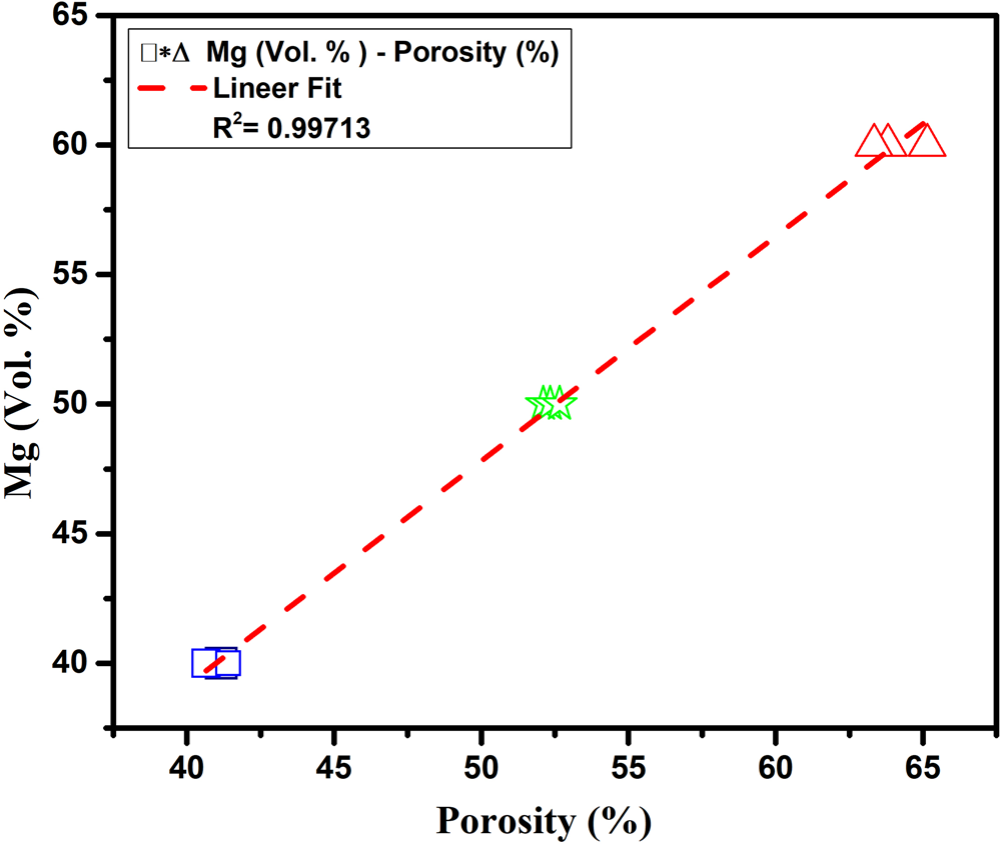
The effect of magnesium additive on porosity

Figure 8 shows the impact of the initial compacts with 40, 50, and 60% Mg on the porosity of the P-Ti6Al4V structures formed through vaporization of Mg. The figure indicates that the total porosity of the P-Ti6Al4V structures exhibited an almost linear increase due to Mg addition (Vol.%) as spacer.

In addition, the mean density and porosity percentages of the P-Ti6Al4V structures are quite consistent with expected values. When porosity reaches to 50% in volume, the density of the sample decreases to 2.11 g/cm^3^, which is slightly less than half of pure Ti6Al4V (4.43 g/cm^3^). As it is known, bone density plays an important role in implantation applications in terms of compatibility with natural bone. The density ranges of the P-Ti6Al4V structures produced in this study confirm that bone density ranges required to keep the bonding and healing ratios between the bone and the implant material as well as providing the low failure rates in implant applications [15, 27]. Moreover, it has been reported in the literature that the ideal porosity of artificial implant or hard tissue biomaterials to be used in biomaterial applications should be between 30% and 90% [10]. The average density and porosity percentage values of the P-Ti6Al4V structures are also consistent with the literature [6, 10, 28–30] outlined in Table 2. An indicator of inter-connectivity between pores is that the number of open pores can be obtained from the total porosity (both open and closed) ratio [31]. This suggests that pores are mostly (over 95%) interconnected regardless of the percentage of porosity, and that interconnections increase with the magnesium powder. This can be explained by the direct impact of Mg particles with large powder size and contact area on pore formation within the Ti6Al4V alloy.

**Table 2:** Comparison of density and porosities of Ti-based structures

Materials	Density (g.cm^−3^)	Porosity (%)	Reference
Cancellous and Corticol bone	(1.4–1.0)–(2.1–1.8)	30–90 and 5–30	[28]
Porous CP Ti	3.01–2.25	33–50	[10]
Porous-Ti6Al4V	–	41–64	[29]
Porous-Ti6Al4V	2.82	36.3	[6]
Porous Ti6Al7Nb	2.1–1.2	53–73	[30]
**This work**	**2.61–1.59**	**41–64**	

**Fig. 9:**
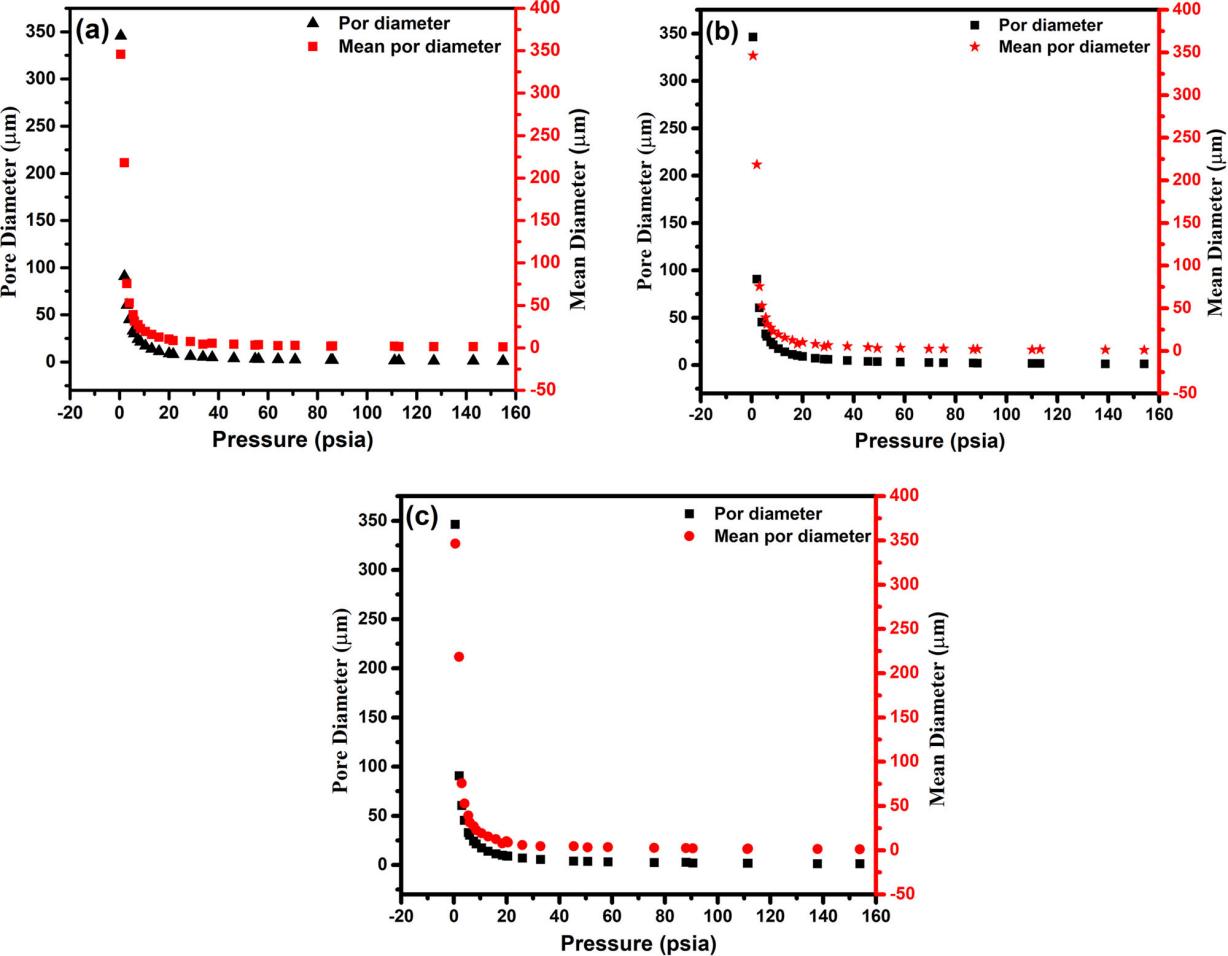
Pore distributions of **a** P-Ti6Al4V (41.08%), **b** P-Ti6Al4V (52.37%), and **c** P-Ti6Al4V (64.10%) structures

Figure 9(a–c) show the results of change in pore size with applied pressure and porosity during the fabrication of P-Ti6Al4V alloys. The mean pore diameters at low pressure (up to 0.52 psi) were recorded as 345.6 µm for 41.08%, 346 µm for 52.37%, and 346.2 µm for 64.10%. From the plots, it is clearly seen that gained values are very close to the initial Mg particle size (average 350 µm). However, the lowest and highest pore diameter values obtained by applying high pressure from 0.52 to 154 psi in order to reveal the presence of smaller sized micropores in P-Ti6Al4V structures were found to be between 1.1 and 90.7 µm. Furthermore, the lowest and highest mean pore size values have been found between 1.1 and 218 µm. The micro and macro pores of Ti6Al4V structures are highly important considering the mechanical anisotropy role of macropores and sufficient vascularization, cell migration as well as nutrient transportation roles of micropores [32] in natural bone structure.

### Microstructural development

**Fig. 10:**
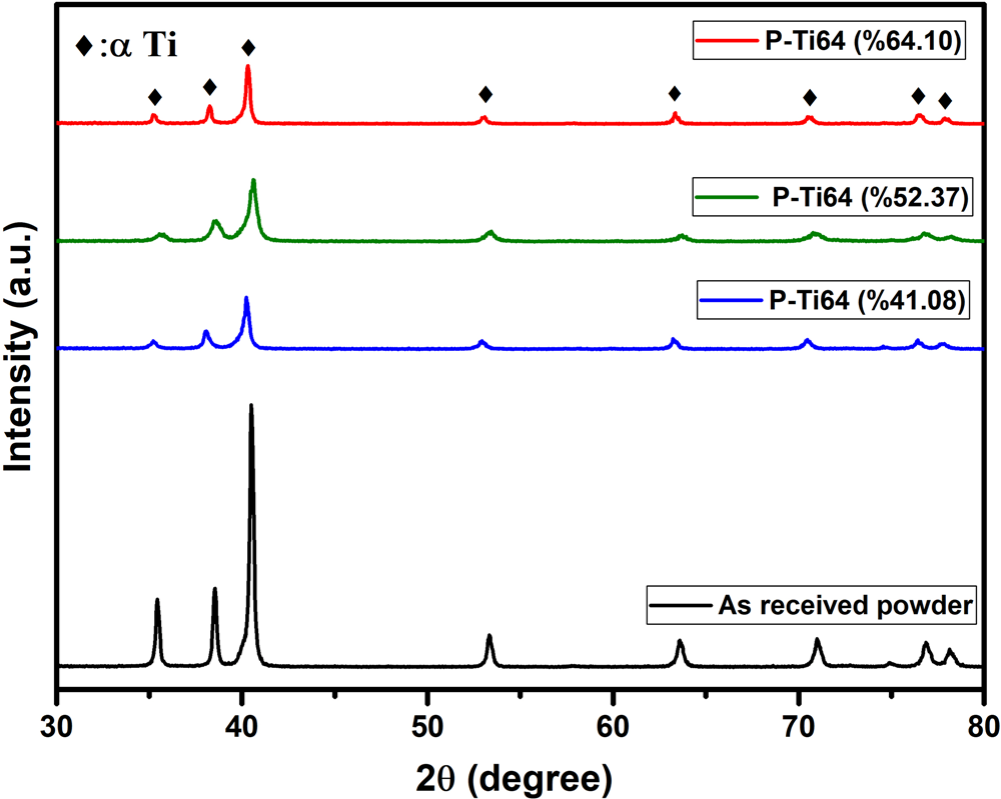
Comparative XRD graphs of as received Ti6Al4V alloy powder, P-Ti6Al4V (41.08%), P-Ti6Al4V (52.37%) and P-Ti6Al4V (64.10%) porous structures

Figure 10 shows the XRD spectra of the used initial Ti6Al4V alloy powder and the produced P-Ti6Al4V alloys. As can be seen from the comparative XRD peaks, neither magnesium and its component used as spacers nor oxidized derivate of titanium (such as TiO, Ti_2_O, Ti_2_O_5_, Ti_3_O_5_) were found in P-Ti6Al4V production. Magnesium is a biocompatible material, which has limited solubility in titanium, can be dissolved in body fluids, and excreted through the excretory system. The effects of Mg presence in bone structure and its contribution to bone ingrowth in biomedical applications have already been reported in the literature [33]. This issue is not discussed here, since trace amount of Mg remained in the structure was assumed that it will not cause any disadvantages, and this phenomenon may require detailed research.

### Mechanical properties

**Fig. 11:**
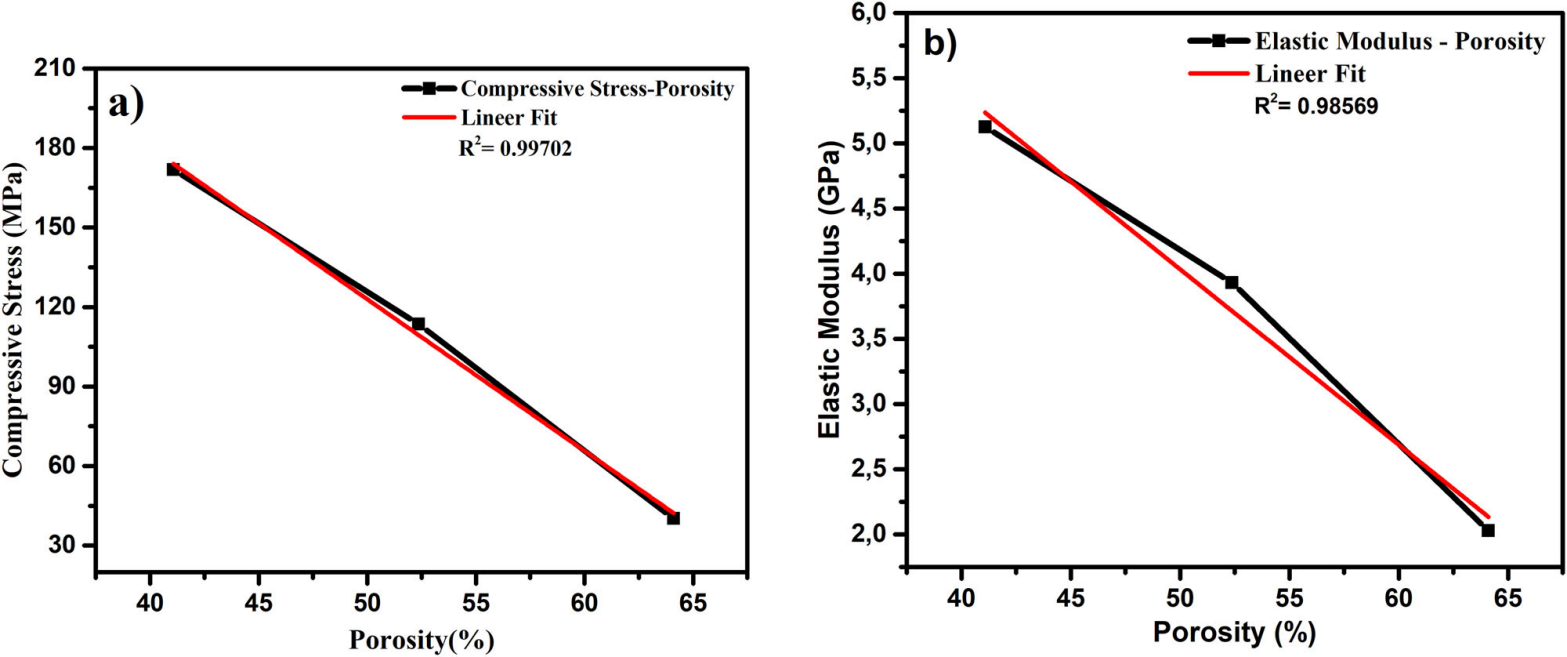
Compression stress (**a**) and elastic modulus (**b**) curves of P-Ti6Al4V structures as a function of porosity

Figure 11a, b respectively show the graphics of the P-Ti6Al4V structures related to compression stress and elastic modulus as a function of porosity. As shown in Fig. 11a, compression strength decreased with increased porosity. Figure 11b shows the elastic modulus values of the P-Ti6Al4V structures with different porosity percentages. The elastic modulus values of the P-Ti6Al4V structures calculated from the slope of the linear elastic zones of the stress and strain curves were found to be 5.13 ± 0.14, 3.93 ± 0.13 and 2.03 ± 0.12 respectively based on the porosity percentages of 41.08, 52.37 and 64.10. As expected, the mechanical tests revealed that the compression strengths and elastic modulus decreased with the increase in porosity. The P-Ti6Al4V structure with 41.08% porosity yielded the highest elastic modulus (5.13 GPa), and strenght (171.86 MPa) value. However, like the relationship between elastic modulus and porosity, a nonlinear relationship beyond the pore size distribution impact exists between porosity and compressive strength.

Table 3 shows a comparison between the compression strength and elastic modulus values of bone along with bulk Ti and the P-Ti6Al4V structures. As it is given, bulk implant materials prevent the necessary stress transfer to bone due to high elastic modulus, and thus; weakening the fixation between the bone and the implant material [34]. In order to eliminate these disadvantages, the use of porous materials with a combination of high strength and low elastic comes to the fore [35]. While the elastic modulus of cancellous bone has values lower than 3 GPa, compact bone values can vary between 1 GPa and 30 GPa. Also, the compression strength of human bone is between 2 MPa and 230 MPa [36, 37].

**Table 3:** Comparative compressive strength and elastic modulus values of the obtained P-Ti6Al4V alloys and natural bone

Materials	Porosity (%)	Compressive strength (MPa)	Elastic Modulus (GPa)	Ref.
Bulk Ti6Al4V	0	840–1100	90–114	[35]
^a^Porous-Ti6Al4V-1	41.08	171.86	5.13	
^a^Porous-Ti6Al4V-2	52.37	113.54	3.93	
^a^PorousTi6Al4V-3	64.10	40.23	2.03	
Porous-Ti6Al4V	45–70	150–28.2	14.7–1.4	[6]
Bone	30–90	230–2	30–1	[36] [37]

^a^The obtained Porous-Ti6Al4V alloys in this study

Various studies have been conducted on mechanical properties of porous Ti and Ti6Al4V structures. Esen and Bor [5, 6] produced Ti and Ti6Al4V alloys with 45 to 70% porosity by using powder magnesium (Mg) as spacer. They determined that the compression strength values of the porous Ti and Ti6Al4V metals ranged from 15 MPa to 116 MPa and from 28.2 MPa to 150 MPa while the elastic modulus values ranged from 0.42 GPa to 8.80 GPa, and from 1.42 GPa to 14.7 Gpa, respectively. Kim et al. [22] determined the compression strengths between 38 to 280 MPa using addition of 50, 60, and 70% Mg. Kalantari et al calculated the compression strength and elastic modulus values of P-Ti6Al4V structures with 47 to 64% porosity depending on the Mg addition as 72 to 132 Mpa, and 37 to 47 GPa, respectively [29]. In the porous Ti study conducted by Chen et al. [10], the compression strengths were determined from 117 to 405 Mpa, and the elastic modulus values were determined from 15.4 to 44.2 GPa. Although the cold pressing and spacer methods were mostly used in the mentioned studies, hot-pressing technique was applied in this study. As a result of current fabrication of P-Ti6Al4V by using hot pressing technique with Mg spacer, obtained values were found to be compatable and consistent with the mechanical values of human bone [37]. Moreover, it is important to declare that the spherical pores were almost homogeneously distributed within the Ti6Al4V structure, and interconnected open pores. When achived results are evaluated, it can be seen that produced P-Ti6Al4V alloys could be a considerable alternative in hard tissue biomedical applications.

## Conclusion

- P-Ti6Al4V alloy have been produced using the hot-pressing technique and spherical magnesium spacers (40, 50 and 60%).
- By using hot pressing technique, homogeneous P-Ti6Al4V alloys were produced at different porosities (41.08, 52.37 and 64.10%) where formed having homogeneous and interconnected pores.
- Porosity increased and density decreased with the increase in the magnesium content.
- The strength and elastic modulus values decreased nonlinear as the porosity increased.
- The compression strengths of the P-Ti6Al4V structures are achieved between 40 MPa and 171 MPa while the elastic modulus values are between 2 GPa and 5 GPa, very close to the elastic modulus of natural bone.
- The production of P-Ti6Al4V structures obtained in the present study with the mechanical values of the bone (compression strength: 2–230 MPa, elastic modulus: 1–30 GPa) may contribute to the harmonious functioning of the bone and implant.
- From the results, it was revealed that using hot pressing technique increase inter-grain sintering and consolidation that can be an alternative potential method to produce P-Ti6Al4V structures for biomedical applications.
